# Demonstration of a New Characterization Method for Weak Measurement

**DOI:** 10.3389/fchem.2022.913035

**Published:** 2022-05-30

**Authors:** Yang Xu, Lixuan Shi, Chongqi Zhou, Zhangyan Li, Tian Guan, Xinhui Xing, Le Liu, Yonghong He

**Affiliations:** ^1^ Shenzhen Key Laboratory for Minimal Invasive Medical Technologies, Tsinghua Shenzhen International Graduate School, Institute of Optical Imaging and Sensing, Tsinghua University, Shenzhen, China; ^2^ Institute of Biopharmaceutical and Health Engineering, Tsinghua Shenzhen International Graduate School, Tsinghua University, Shenzhen, China; ^3^ Department of Physics, Tsinghua University, Beijing, China; ^4^ School of Medicine, Tsinghua University, Beijing, China; ^5^ Shenzhen International Graduate School, Institute of Materials Research, Tsinghua University, Shenzhen, China

**Keywords:** weak measurement, molecular hydrolyzed, characterization methods, transition conditions, measuring range

## Abstract

In this work, the difference between the weak measurement method and the weak value amplification process and the classical measurement process is thoroughly discussed, and the transition conditions of the weak value enhancement are obtained. A transition mode of the weak measurement and the classical measurement is proposed for the first time, and a better fitting model of the measurement results is found by performing a systematic analysis. On top of that, the importance of the new fitting method for the application of the weak measurement system is verified during the industrial production of organic molecular -nucleic acid, protein, polysaccharide-hydrolysis or synthesis. At the same time, a variety of spectral characterization methods are proposed and the advantages and disadvantages of the different characterization methods are analyzed through carrying out experiments. Consequently, the wide implementation of weak measurement-based detection technology is attained.

## 1 Introduction

The concept of the weak measurement was proposed in 1988 by Aharonov, Albert and Vaidman (AAV) on the basis of “two-state vector representation of quantum mechanics” (Aharonov et al., 1988; Ritchie et al., 1991). In 2005 and 2007, the teams of Pryde and Jozsa realized and measured complex weak values in polarization detection based on the weak measurement theory and explained in detail the physical significance of the weak values in both the real and imaginary parts of the actual measurement (Pryde et al., 2005; Jozsa, 2007). With this approach, it was possible to achieve high precision measurement with weak measurement technology (Xu et al., 2013; Vella et al., 2019; Xu et al., 2020; Yin et al., 2021). Since 2010, relevant theories have shown that the weak measurement technology exhibits more obvious detection advantages in the frequency domain than the other fields (Brunner and Simon, 2010; Xu et al., 2013). In 2015, our group first proposed a novel optical frequency domain weak measurement system with universal value, which has shown amazing potential in the field of biomolecular detection (Zhang et al., 2016; Li et al., 2017; Li et al., 2018; Xu et al., 2018; Xu et al., 2019a; Xu et al., 2019b; Xu et al., 2021).

Since it was put forward, the weak measurement method has been considered a theoretical scheme of indirect measurement by means of pointer coupling with pre-selected system states. However, the amplification effect of the weak measurement relies on the theory of quantum measurement and has been widely discussed in many aspects for a long time. Even some fundamental properties of the quantum measurement itself have been debated and unsolved since its birth. Along these lines, Von Neumann’s mathematical description is considered the theoretical basis of quantum measurement (Von Neumann, 2018). It was assumed that the measurement process could be regarded as the measurement of the probability of the quantum operator A in each state, which is the measurement, where. Compared with the classical measurement approach, the weak measurement method proposes the utilization of a weak measurement quantum system with a weak value amplification effect, which is induced by introducing a post-selection process. More specifically, when the weak value decreases, the probability of the post-selection is very low, but the amplification effect of the measurement system is greatly improved (Hosoya and Shikano, 2010; Pusey, 2014; Avella et al., 2017). The proposed theory has been verified in many experiments since then (Hosten and Kwiat, 2008; Dixon et al., 2009; Jordan et al., 2014), and has been also successfully implemented in a variety of fields (Aharonov et al., 2003; Kocsis et al., 2011). The difference between the weak measurement and the strong measurement method is theoretically considered to be the strength of the interaction between the measurement system and the measurement object. On the other hand, the use of weak values is often considered to be the fundamental difference between the weak and strong measurements, especially in the experimental-related work since the weak and expected values are different both in concept and in the extracted measurement results (Duck et al., 1989; Dziewior et al., 2019).

Therefore, the quantitative description of the difference between the weak and strong measurements, and even the realization of the transition between the weak and strong measurements in the experiment, becomes particularly important. PAN, Y successfully realized the continuous transition scheme from weak to the strong measurement of a bound 40Ca + single atom, while it was assumed that the transition process can be expressed by the characteristic index (Pan et al., 2020). This transformation of the continuous connection between the weak and strong measurements opens up new experimental possibilities for testing the basis of quantum measurement, and also renders the improvement of the measurement scheme of the related quantum technology of vital importance.

Under this direction, in this work, the premise of the weak value amplification is systematically examined. In addition, the significance of the weak measurement procedure itself is explored, as well as the fundamental difference between the weak and classical measurement approaches. By discussing the approximate range of weak values, a unified equation to the description of both of them is provided, whereas the principle of the weak value enhancement effect and the significance of weak value for the weak value amplification process is examined providing a possible way of the enhancement. The reliability of the proposed theory is also proved in the frequency domain, space domain and electronic case, and the acquired experimental results are proved in the frequency and space domains. A new fitting method is established by using the new descriptive equation, which can smoothly provide the transition to the classical measurement state.

## 2 Theory

H=−g(t)PA (1)

The discussion of this work still considers the coupling Hamiltonian as the starting point, and the eigenvalues of 1 and -1 as the measurement results of the orthogonal photon polarization operator A are used. The coupled Hamiltonian satisfies the following equation:where g(t) represents the time-dependent coupling strength, satisfying the following condition: g(t)dt=k . k stands for the coupling strength of the system state and the readout pointer state; P is the intrinsic state of the photon (which can represent the transverse distribution of the photon or the photon momentum distribution). In the case of post-selection, the eigenstate can be expressed as follows:

$$\begin{array}{l} \langle \psi_{f}\left|e^{-i\int Hdt}\right|\psi_{i}\rangle \exp\left(-\frac{P^{2}}{4{\left(\text{Δ}P\right)}^{2}}\right)=\langle \psi_{f}|\psi_{i}\rangle \sum_{n=0}^{\infty }\frac{{\left(iP\right)}^{n}}{n!}{\left(A^{n}\right)}_{\omega }\exp\left(-\frac{P^{2}}{4{\left(\text{Δ}P\right)}^{2}}\right)\\ =\langle \psi_{f}|\psi_{i}\rangle \exp\left(iP\frac{\langle \psi_{f}\left|\hat{A}\right|\psi_{i}\rangle }{\langle \psi_{f}|\psi_{i}\rangle }\right)\exp\left(-\frac{P^{2}}{4{\left(\text{Δ}P\right)}^{2}}\right)\\ =+\langle \psi_{f}|\psi_{i}\rangle \sum_{n=0}^{\infty }\frac{{\left(iP\right)}^{n}}{n!}\left[{\left(A^{n}\right)}_{\omega }-{\left(A_{\omega }\right)}^{n}\right]\exp\left(-\frac{P^{2}}{4{\left(\text{Δ}P\right)}^{2}}\right) \end{array}$$ (2)

In the weak measurement-related discussions, the second term of the above equation is usually omitted to ensure the validity of the weak value description of the optical path system (Aharonov and Vaidman, 1990). It is generally believed that this approximation needs to meet the following precondition:

$${\left(2\Delta P\right)}^{n}\frac{\Gamma \left(n/2\right)}{\left(n-2\right)!}\left|{\left(A^{n}\right)}_{\omega }-{\left(A_{\omega }\right)}^{n}\right|\ll 1$$ (3)

The approximate condition is accepted by most of the weak measurement-related theories (Aharonov et al., 1988; Ritchie et al., 1991; Brunner and Simon, 2010; Xu et al., 2013; Zhang et al., 2016), but the constraint is not natural. A photon dimorphic system is considered as follows: only two states exist in the input light, and the pre- and post-selective states of the photon can be expressed as follows:

$$\begin{array}{l} |\psi_{i}\rangle =\sin\alpha |H\rangle +\cos\alpha |V\rangle \\ |\psi_{f}\rangle =e^{-i\delta }\cos\beta |H\rangle -e^{i\delta }\sin\beta |V\rangle \end{array}$$ (4)

The above equations represent the polarization angle of the selected states before and after, respectively, and denote the phase difference to be measured. At this point, since the system pointer is selected as the photon wavelength, the system Hamiltonian can be expressed by the following expression:

$$H=-k\left(\hat{\lambda }-\lambda_{0}\right)\hat{A}$$ (5)

$\lambda -\lambda_{0}$ is selected as the pointer state operator and $\hat{A}=|HH|-|VV|$ asthe observation operator of the system. When the incident light is $e^{\frac{{\left(\lambda -\lambda_{0}\right)}^{2}}{\sigma_{\lambda }^{2}}}$ , $\lambda_{0}$ is the central wavelength of the incident light spectrum, and ${\text{σ}}_{\text{λ}}$ is the standard deviation of a Gaussian wave, and in calculations, we use the half-peak width for approximation. At this point, the pointer state after passing the weak measurement system can be represented as follows:

$$\begin{array}{l} |\varphi_{o}\rangle =\langle \psi_{f}|e^{-ikA\left(\lambda -\lambda_{0}\right)}|\psi_{i}\rangle |\varphi_{i}\rangle \\ =\left[\sin\left(\alpha -\beta \right)\cos\delta -i\sin\left(\alpha +\beta \right)\sin\delta \right]e^{-\frac{{\left(\lambda -\lambda_{0}\right)}^{2}}{\sigma_{\lambda }^{2}}}|\lambda \rangle \end{array}$$ (6)

The weak value is defined as (Li et al., 2018; Xu et al., 2021):

$$\begin{array}{l} A_{\omega }=\frac{\sin\left(\alpha +\beta \right)\sin\left(\alpha -\beta \right)}{\sin^{2}\left(\alpha +\beta \right)\sin^{2}\delta +\sin^{2}\left(\alpha -\beta \right)\cos^{2}\delta }\\ +i\frac{\left[\sin^{2}\left(\alpha +\beta \right)-\sin^{2}\left(\alpha -\beta \right)\right]\cos\delta \sin\delta }{\sin^{2}\left(\alpha +\beta \right)\sin^{2}\delta +\sin^{2}\left(\alpha -\beta \right)\cos^{2}\delta } \end{array}$$ (7)

Because the constraints need to be satisfied, the value of ${\left(A^{n}\right)}_{\omega }$ has to be calculated.

$${\left(A^{n}\right)}_{\omega }=\frac{\langle \psi_{f}|\hat{A}|\psi_{i}\rangle }{\langle \psi_{f}|\psi_{i}\rangle }=\frac{\sin\alpha \cos\beta e^{-i\delta }-{\left(-1\right)}^{n}\cos\alpha \sin\beta e^{i\delta }}{\sin\alpha \cos\beta e^{-i\delta }-\cos\alpha \sin\beta e^{i\delta }}=\{\begin{array}{c} 1,n=2m,m=1,2,3,\ldots \\ A_{\omega },n=2m+1,m=1,2,3,\ldots \end{array}$$ (8)

When ${\left(A^{n}\right)}_{\omega }$ does not converge with n it is implied that the constraints of Eq. 3 should be changed to $\left|A_{\omega }\right|\ll 1$ and Δq≪1 . In general, the initial state of the optical path satisfies the following condition: α,β≪1 , which does not always make the constraint hold. The constraints for the fore and the aft polarization states need to be modified to α≠β and b≪1 . If the condition of α≠β is not satisfied, the spectral form of the double peaks will appear (although it does not meet the phenomenon of Gaussian center “offset” that was mentioned in AAV theory, it can still calculate the center of the double peaks to measure the “offset”). If the condition of b≪1 is not satisfied, the phase change only affects the light intensity, but the shift of the central wavelength is not affected. Thus, the law of the weak measurement system is consistent with that of the classical measurement. Therefore, the premise of the approximation condition of the weak value description is that the pre-and post-selection states are not orthogonal and the readout spectrum has a direct flow. In fact, the form is closer to the motion of the spectral wave packet. At the same time, because $A_{\omega }$ cannot be infinitely enlarged, the impact of the weak value amplification cannot be infinitely enlarged.

The change of the spectrum when the phase difference of the optical path is adjusted is basically consistent with the change of the experimental measurement, but there are still some special cases. In one case, the fitting effect is not good when the initial optical path difference is very large. At this point $ReA_{\omega }\gg 1$ , and the system can be considered as a classical measurement form. In the other case, the offset cannot increase infinitely, which corresponds to $ImA_{\omega }\gg 1$ . Consequently, the fitting condition of the weak value amplification is not met at this time, but the description method of the weak value is still applicable. Hence, the weak measurement establishment condition in AAV theory can only fit a certain part of the corresponding curve well.

**FIGURE 1:**
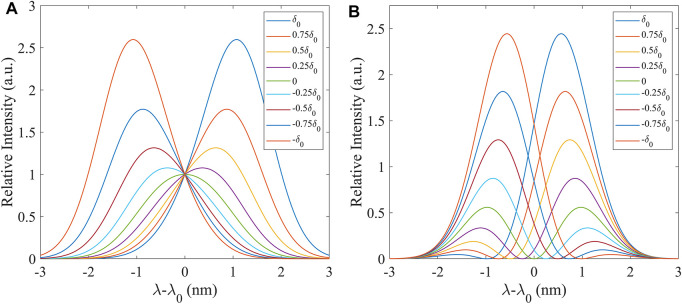
Theoretical spectra of the different phase differences under the enforcement of weak measurement conditions. The spectrum with different colors represents the various phase differences in the light path. **(A)** The α≠β ideal case is described in AAV theory. **(B)**  α=β or α is often close to β .

When the post-selection state of the system is set to a specific state, the displacement of the pointer far exceeds the eigenvalue of the system in the interaction Hamiltonian of the weakly coupled pointer measurement. In addition, under the first-order approximation, the displacement of the center of the pointer state is proportional to both the coupling parameter and the weak value. Figure 1 shows the spectral migration modes of the pre- and post-selection states under the application of the non-orthogonal and near-orthogonal conditions respectively. The theoretical curve described in AAV theory is shown in Figure 1A, which represents the offset of the Gaussian distribution spectrum. Figure 1B illustrates a more common result when the system’s pre- and post-selection states are in a nearly orthogonal combination. The spectrum is found in the form of two peaks, and when the post-selection state changes, the two peaks decrease in the opposite direction. This law has been well studied in-depth in past work (Zhang et al., 2016; Li et al., 2018; Xu et al., 2018), while the latter mode is generally selected in the experiments to obtain both better measurement range and resolution.

For the sake of discussion, let $x=\lambda -\lambda_{0}$ represent the narrow band width of the spectrum and let $y=I_{rec}\left(x\right)$ be the distribution function of the spectrum. On the premise of δ≪1 and α≈β , we can obtain the following form:

$$y=\left[1-cos\left(kx+\delta \right)\right]e^{-x^{2}/\sigma_{\lambda }^{2}}$$ (9)

We now need to estimate the phase difference information from the measured spectral y(x) relation plot. The theoretical spectrum given by Eq. 9 can be obtained from the maximum likelihood estimate (Strubi and Bruder, 2013).

$$\sum_{i}\left(1-cos\left(kx_{i}+\delta \right)\right)sin\left(kx_{i}+\delta \right)e^{-2x_{i}^{2}/\sigma_{\lambda }^{2}}=\sum_{i}y_{i}sin\left(kx_{i}+\delta \right)e^{-x_{i}^{2}/\sigma_{\lambda }^{2}}$$ (10)

In the above formula, $x_{i}$ and $y_{i}$ represent the normalized wavelength and the corresponding normalized light intensity distribution respectively. At this point, another constraint needs to be considered: kx≪1 , which implies that the product of the coupling constant and the measurement bandwidth is relatively small. Therefore, δ can be divided into the following two forms:

1) If δ≫kx , then $kx_{i}+\delta \approx \delta$ , and Eq. 10 can be simplified as follows:

$$1-\text{cos}\delta =\frac{\sum e^{-x_{i}^{2}}y_{i}}{\sum e^{-2x_{i}^{2}}}$$ (11)

2) If δ,kx≪1 , then $kx_{i}$ cannot be ignored, but $kx_{i}+\delta \ll 1$ still exists. In this case, Eq. 10 can be simplified as follows:

$$\tan\delta ==\frac{k\sum e^{-x_{i}^{2}}x_{i}y_{i}}{\sum e^{-x_{i}^{2}}y_{i}}$$ (12)

In the first case, the phase difference that has to be measured is relatively large. Therefore, the right-hand side of the equation represents the ratio of the received light intensity to the initial light intensity $\sum y_{i}$ and the ratio of the outgoing light intensity $\sum e^{-2x_{i}^{2}}$ . In the second case, the right side of the equation represents the shift of the central wavelength $\sum x_{i}y_{i}/\sum y_{i}$ after multiplying the weight of the light intensity $\sum e^{-x_{i}^{2}}$ , whereas the coupling intensity K needs to be considered as the coupling amplification factor. This situation is generally consistent with the previously reported weak measurement theory, but the initial spectral intensity should be considered as the weight of the central wavelength shift. When δ=0 , the measurement sensitivity is proportional to 1/K in case 2 and to 0 in case 1. Because the value of K is relatively small, the measurement sensitivity of case 2 is far higher than that of case 1 at this time, and the amplification effect of the weak value can be reflected at this time. A similar conclusion can be obtained for the non-Gaussian incident light, but the initial light intensity distribution term in Eqs 11, 12 needs to be modified.

The transition from weak to classical measurements can be now further obtained. If the above equation is expanded with respect to $kx_{i}$ and δ , the following equation is derived:

$$\sum_{i}\left(1-cos\left(kx_{i}+\delta \right)\right)tankx_{i}e^{-2x_{i}^{2}/\sigma_{\lambda }^{2}}+\sum_{i}\left(1-cos\left(kx_{i}+\delta \right)\right)tan\delta e^{-2x_{i}^{2}/\sigma_{\lambda }^{2}}=\sum_{i}y_{i}tankx_{i}e^{-x_{i}^{2}/\sigma_{\lambda }^{2}}+\sum_{i}y_{i}\tan\delta e^{-2x_{i}^{2}/\sigma_{\lambda }^{2}}$$ (13)

We note that due to the first-order approximation, in case 1 the first term on both sides of Eq. 13 is ignored, and in case 2 the left side of the equation is completely ignored. Therefore, in order to obtain an equation that can satisfy the transition process, the first term on the left side of the equation can be ignored, and the remaining three terms of the equation, under the constraint condition $kx_{i}\ll 1$ , can be obtained as follows:

$$\frac{2z^{2}}{1+z^{2}}\sum_{i}e^{-2x_{i}^{2}/\sigma_{\lambda }^{2}}-\sum_{i}e^{-x_{i}^{2}/\sigma_{\lambda }^{2}}y_{i}=\frac{2z}{1-z^{2}}\sum_{i}e^{-x_{i}^{2}/\sigma_{\lambda }^{2}}y_{i}kx_{i}$$ (14)

where z=tan⁡δ/2 . At this point, an implicit function can be used for the transition from the weak measurement state to the classical measurement state, where the phase difference can be predicted from the spectrogram.

So far, a calculation function independent of the weak value has been obtained, but the influence of the weak value amplification can be still achieved. Since the weak measurement method has an amplification effect, the source of the amplification is weak to the coupling mode k, and the weak value is only a representation form of the amplification effect. For example, b≪1 and $ImA_{\omega }\approx \frac{\left({sin}^{2}\left(\alpha +\beta \right)-{sin}^{2}\left(\alpha -\beta \right)\right)ranb}{{sin}^{2}\left(\alpha -\beta \right)}\propto \tan b\propto b$ , the central wavelength can be represented by the following expression: ImAω , by considering the second-order Taylor approximation:

tan⁡b=∑e−xi2⁡tan(kxi)yi∑e−xi2yi∝ImAω (15)

At this point, we can argue that ImAω is a better approximation under this premise. Therefore, the following expression is still valid and can be used: dλ∝ImAω . According to the AAV theory, weak values occur during the interaction coupling process as can be ascertained from the following equation:

$$|\varphi_{o}\rangle =\langle \psi_{f}|e^{-gAx}|\psi_{i}\rangle |\varphi_{i}\rangle =\left[\sin\left(\alpha -\beta \right)\cos b-i\sin\left(\alpha +\beta \right)\sin b\right]e^{-x^{2}}e^{-gA_{\omega }x}$$ (16)

Obviously, the theory of AAV is a general framework, which represents the strength of the mutual coupling between the measurement operator and the coordinate space. However, if the measurement operator A is acting on mutually orthogonal eigenstates, it can be directly calculated without using the AVV. In this case, since $ImA_{\omega }\propto \tan b$ can be used as the description of the phase under the second-order Taylor approximation, and ImAω can measure the constraint of $\sin^{2}\left(\alpha -\beta \right)\ll 1.$ Besides, it can also measure the weak value amplification effect of the system. However, the sensitivity enhancement of the weak values is relatively limited (Aharonov et al., 1988; Ritchie et al., 1991; Xu et al., 2013), because the approximation conditions of AAV are not satisfied at $ImA_{\omega }\gg 1$ . Therefore, the theory of the weak value amplification is not valid beyond a certain measurement range. In addition, the employed Gaussian light may not be completely normal in the experiment, so the waveform of the incident light needs to be considered for the weight calculation during the fitting procedure.

## 3 Experiment

### 3.1 A Comparison of Multiple Characterisation Methods for Weak Measurements

**FIGURE 2:**
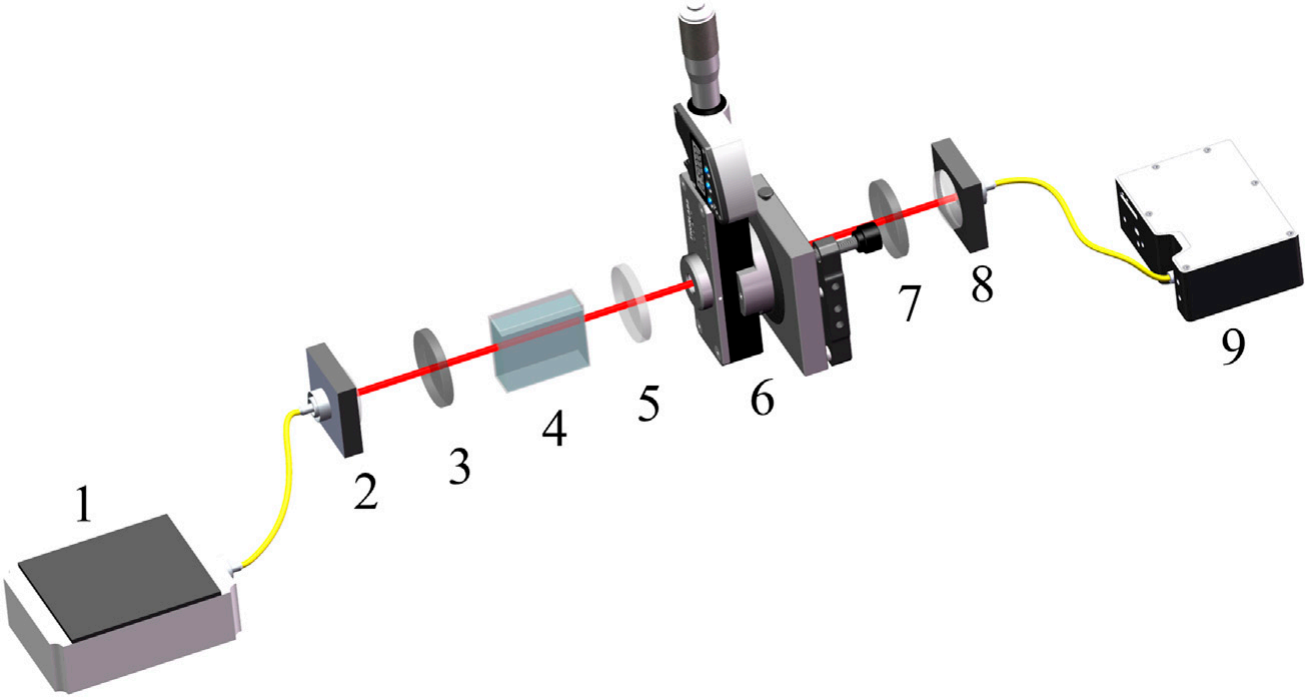
Schematic installation of the weak measurement sensor. 1, Superluminescent laser diode (SLD, IPSDD0804, 5 mW, Center wavelength: 840 nm, bandwidth: 40 nm, Inphenix). 2 and 8, Collimating and coupling lenses. 3 and 7, Pre- and post-selection polarizer (Thorlabs Inc., 180 LPVIS050-MP, extinction ratio of 100,000:1). 4, Sample Cell. 5, Achromatic Quarter Wave Plate (Thorlabs Inc., AQWP05M-980). 6, Soleil-Babinet Compensators (SBC, Thorlabs Inc., SBC-IR). 9, Spectrometer (Ocean Optics, HR4000).

The experiments in this work were carried out by using an optical rotation weak measurement system (Li et al., 2018). As is shown in Figure 2, an SLD was selected as the light source. After collimation is performed through the coupling lens, the light beam entered the weak measurement system that was coupled to the spectrometer to read the spectral signal. Additionally, the weak measurement system consists of a front selective polarizer, a wave plate and Soleil-Babinet Compensators (which provide the initial phase difference), a sample cell and a rear selective polarizer. In the experiment, only distilled water was injected into the sample cell to simulate the actual measurement scene, and the Soleil-Babinet Compensators were adjusted to simulate the phase difference of the circularily polarized light generated in the system.

Since Eq. 13 requires that the phase difference estimation should be performed by using weighted central wavelength offset, this approach is inconsistent with the requirements of the AAV theory. At the same time, since the outgoing spectrum of the SLD light source does not completely conform to the Gaussian distribution, and the transmittance of components may be affected in the transmission process, resulting in the manifestation of spectral morphology changes, the influence of the non-Gaussian spectrum distribution on measurement should also be considered. In order to compare the advantages and disadvantages of these methods, five different wavelength-processing methods were selected for the phase difference estimation, while their measurement range and resolution are analyzed. The five treatment methods are as follows:

A. Based on our previous work (Zhang et al., 2016; Li et al., 2017; Li et al., 2018), $\text{δ}\propto I_{m}A_{m}$ ; B. This can be approximated to $\text{δ}\propto \frac{\Sigma e^{-\lambda^{2}}\lambda \varphi \left(\lambda \right)}{\Sigma e^{-\lambda^{2}}\varphi \left(\lambda \right)}$ according to Eq. 12. here we assume a Gaussian distribution for φ(λ) . $\text{φ}\left(\text{λ}\right)=e^{-\frac{{\left(\lambda -\lambda_{0}\right)}^{2}}{\sigma_{\lambda }^{2}}}$ , where ${\text{λ}}_{0}$ is the central wavelength and ${\text{σ}}_{\text{λ}}$ is the standard deviation of the wavelength (here we use the spectral half-peak width approximation). When the central wavelength is calculated, the theoretical intensity of 840 and 40 nm half peak width is weighted;

C. According to Eq. 12, $\text{δ}\propto \frac{\Sigma e^{-\lambda^{2}}\lambda \varphi \left(\lambda \right)}{\Sigma e^{-\lambda^{2}}\varphi \left(\lambda \right)}$ , considering that the spectral shape of the original spectrum is a skewed Gaussian distribution, we assume $\text{φ}\left(\text{λ}\right)=\{\begin{array}{c} e^{-\frac{{\left(\lambda -\lambda_{0}\right)}^{2}}{\sigma_{\lambda_{1}}^{2}}\;\lambda <\lambda_{0}}\\ e^{-\frac{{\left(\lambda -\lambda_{0}\right)}^{2}}{\sigma_{\lambda_{2}}^{2}}\;\lambda >\lambda_{0}} \end{array}$ and use Newton’s method to approximate ${\text{σ}}_{{\text{λ}}_{1}}$ , ${\text{σ}}_{{\text{λ}}_{2}}$ .

D. According to Eq. 12, $\text{δ}\propto \frac{\Sigma e^{-\lambda^{2}}\lambda \varphi \left(\lambda \right)}{\Sigma e^{-\lambda^{2}}\varphi \left(\lambda \right)}$ , φ(λ) is given directly from the original spectrum.

E. According to Eq. 14, we solve directly for δ

In A, B, C, D, and E, δ indicates the phase difference to be measured.

**FIGURE 3:**
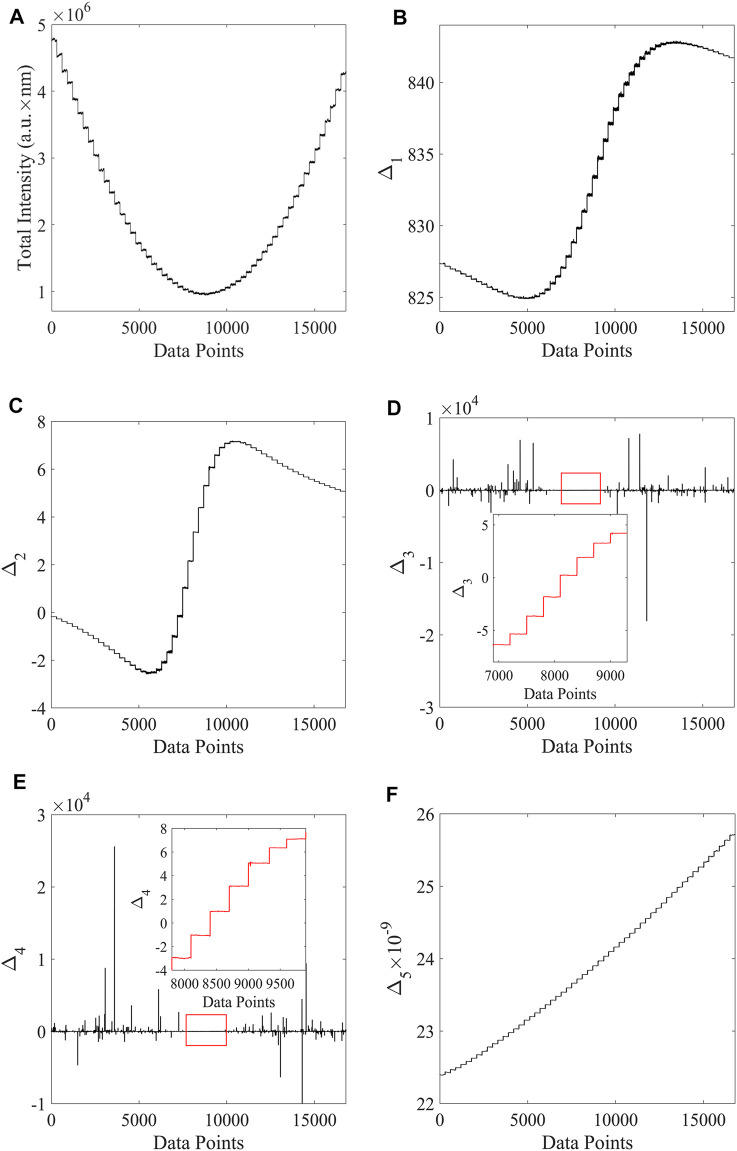
**(A)** Distribution of the spectral relative light intensity corresponding to different phases. **(B–F)** Offsets and phase diagrams corresponding to the five different characterization methods A, B, C, D, **(E)**

The measurement results are shown in Figure 3, and the measurement range and resolution are summarized in Table 1.

**TABLE 1:** Comparison of the measurement effects of various characterization schemes.

Characterization Methods	Resolution (rad)	Measurement Range (rad)
Method A	5.6 × 10^–6^	0.042
Method B	1.6 × 10^–6^	0.024
Method C	0.9 × 10^–6^	0.018
Method D	1.2 × 10^–6^	0.015
Method E	7.2 × 10^–6^	0.168

The system was firstly adjusted to the vicinity of the bimodal working area (Zhang et al., 2016; Xu et al., 2018). As is shown in Figure 3, the spectra of the system were obtained at different optical rotation angles through the SBC. For every 300 data points recorded during the measurement, the rotation angle in the optical path was changed by 0.003 rad by SBC. Figure 3A: Relative light intensity corresponding to the different optical rotation angles. Figures 3B– Method A: No weighting. Figures 3C– method B: weighted with a central wavelength of 840 nm and half peak width of 40 nm. Figures 3D– Method C: Perform skewness fitting merge weighting calculation. Figures 3E– Method D: Weighted calculation by original spectrum. Figures 3F– Method E: In order to achieve a wider measurement range and transition from the weak to strong measurements, this problem was solved by using the transition Eq. 16.

Under this perspective, the measurement range of the above fitting methods was firstly compared. According to the conclusion of the existing work, the weak value amplification range near the spectrum appears as a bimodal state. Furthermore, there is a sensitive range by adjusting the polaroid and the wave plate group before and after the weak value enlarged working range is derived. By working in the same range, and selecting a continuous adjustment of 0.003 rad as the step for the phase difference, the spectrum data changes over time were recorded. The measurement range of the fitting method is the linear relationship between the spectral characterization results and the optical rotation Angle. Then, the resolution of the system under different fitting methods was calculated by employing the following formula: $\delta \lambda /\delta \alpha \sigma_{\alpha }=3\sigma_{s}/\left(\delta \lambda /\delta \alpha \right)$ . As is shown in Table 1, the resolution obtained by the central wavelength shift-A scheme is 5.6 × 10^–6^ rad, and the linear measurement range is 0.036 rad. The resolution was 1.6 × 10^–6^ rad with the weighted central wavelength shift-B scheme, and the measurement range was reduced to 0.024 rad. The resolution of skewness Gaussian fitting-C scheme was 0.9 × 10^–6^ rad and the measurement range was 0.018 rad, while the resolution of the original spectral fitting-D scheme was 1.2 × 10^–6^ rad and the measurement range was 0.015 rad. The extracted results show that the weighted center wavelength has a higher resolution and a narrower linear measurement range. In addition, if a non-Gaussian initial spectrum is considered for the fitting process, the resolution will be improved. The resolution can also be improved to some extent by using the original spectral weight during the fitting process. However, this method means that the measurement range will be narrower than the original scheme proposed by the AAV. However, compared with the classical measurement method, the method of solving the equation-E scheme has a wider linear region, while the measurement range is greatly increased to 0.168 rad, but the detection accuracy is reduced to 7.2 × 10^–6^ rad.

### 3.2 Sucrose Hydrolysis Was Monitored by a Weak Measurement System, and Characterised by Different Methods

Fructose is the sweetest monosaccharide in nature. It is interesting to notice that its sweetness is about 1.8 times that of sucrose and 3 times that of glucose. Fructose will not only cover up the flavor of food, but also has the effect of enhancing the flavor of food. On top of that, it possesses greater solubility and faster dissolution than sucrose and glucose, so it is widely used in the beverage and food industry. At present, monosaccharides are mainly produced by hydrolysis of starch or sucrose in industrial production. Since it is very important to monitor the sugar production process, and because the raw materials and products of the sugar production have optical rotation, the product transformation procedure can be monitored by controlling the optical rotation change in the reaction tank during the implementation of the hydrolysis sugar production process.

$$C_{\mathrm{12}}H_{\mathrm{22}}\mathrm{O11+}H_{2}O\overset{H^{+}}{\to }C_{6}H_{\mathrm{12}}O_{6}+C_{6}H_{\mathrm{12}}O_{6}$$ (17)

**FIGURE 4:**
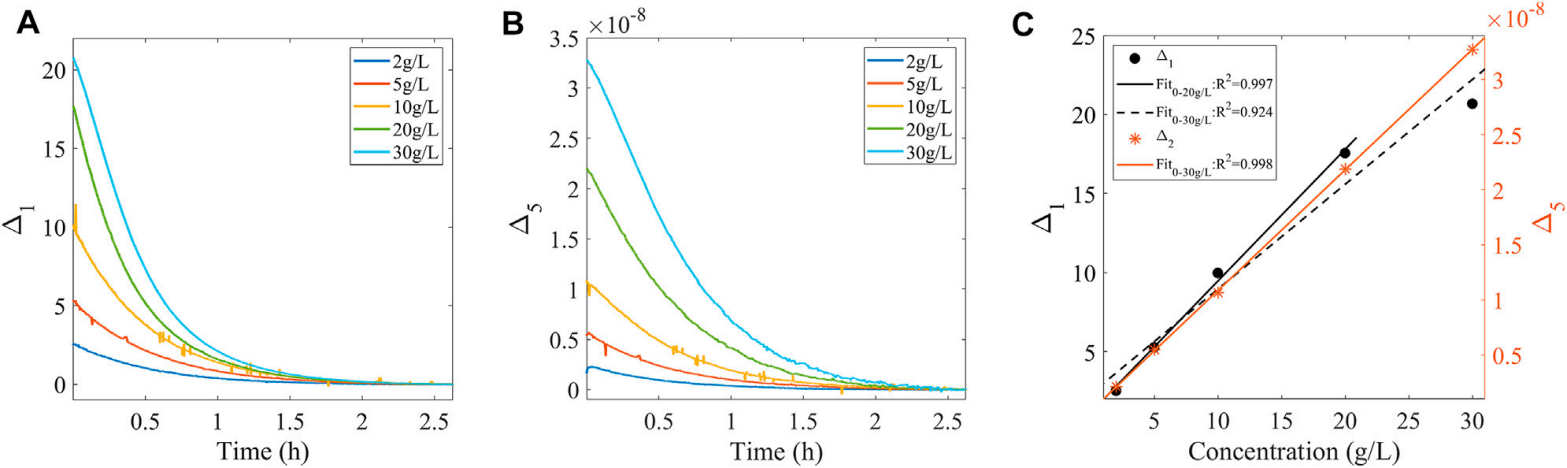
**(A,B)** Characterization results of the hydrolysis process of sucrose for Methods A and E, respectively. **(C)** Linear fit of the two characterization methods corresponding to the amount of change in the hydrolysis process for different concentrations of the sucrose solutions.

The weak measurement system in Figure 2 was used for the sucrose hydrolysis experiment, and the reaction equation is shown in (17). More specifically, sucrose solutions with concentrations of 2, 5, 10, 20, and 30 g/L were placed in the sample tank in Figure 2, and an appropriate amount of hydrochloric acid was added for the hydrolysis process. Meanwhile, the spectral changes during hydrolysis were monitored. As is shown in Figures 4A,B, methods A and E were used to characterize the hydrolysis process, respectively (methods B, C, and D were not considered here due to their small measurement range). As is shown in Figure 4C, when method A was used to characterize the sucrose hydrolysis process, good linearity in the range of sucrose concentration 0–20 g/L was demonstrated, but it was decreased in the range of sucrose concentration 0–30 g/L. However, when represented by method E, good linearity was always acquired. Many industrial processes, such as hydrolyzed starch, sucrose to sugar, hydrolyzed protein to the polypeptide, are carried out under the manifestation of high concentration conditions, accompanied by a wide range of rotation of the reactants. Therefore, for practical scenarios such as industrial production, method E is considered more suitable.

## 4 Conclusion

In this work, the premise of weak value amplification was discussed, and the improvement scheme of the weak measurement method was proposed, as well as the difference between the weak value amplification and the classical measurement processes and the transition conditions. The premise of the weak value amplification is that the form of pre- and post-selection states is close to or completely orthogonal, and the weak coupling condition of pre- and post-selection states is required, which is consistent with the conclusion of other works (Leggett, 1989; Peres, 1989). In addition, aiming at the narrow measurement range of the weak measurement in the frequency domain, a transition scheme between the weak measurement and the classical measurement is proposed for the first time, and a better fitting model of the measurement results was found. In addition, insights from the sucrose hydrolysis experiments confirmed that this model has more advantages than the other characterization schemes in a large range of detection scenarios. Under the application of the classical conditions, the measurement effect of this transition measurement method is no different from that of the polarimeter. Under the premise of the weak value amplification, the measurement results obtained by the new transition equation can extend the application range of the weak measurement and realize the transition with the classical measurement method. Interestingly, the fittingmethod is not affected by the experimental device, and can be applied to many measurement fields of weak measurement in the frequency domain, such as biomolecular measurement, chiral molecular measurement, chiral material detection, and temperature detection, etc.

## Data Availability

The original contributions presented in the study are included in the article/Supplementary Material, further inquiries can be directed to the corresponding authors.
